# Correction: Ghoreyshi, S.M.; et al. Effects of Dietary Supplementation of L-Carnitine and Excess Lysine-Methionine on Growth Performance, Carcass Characteristics, and Immunity Markers of Broiler Chicken. *Animals* 2019, *9*, 362

**DOI:** 10.3390/ani9090608

**Published:** 2019-08-26

**Authors:** Seyed Mohammad Ghoreyshi, Besma Omri, Raja Chalghoumi, Mehrdad Bouyeh, Alireza Seidavi, Mohammad Dadashbeiki, Massimo Lucarini, Alessandra Durazzo, Rene van den Hoven, Antonello Santini

**Affiliations:** 1 Department of Animal Science, Rasht Branch, Islamic Azad University, Rasht 41335-3516, Iran; 2 Laboratory of Improvement and Integrated Development of Animal Productivity and Food Resources, Department of Animal Science, College of Agriculture of Mateur, University of Carthage, Bizerte 7000, Tunisia; 3 Department of Veterinary Science, Rasht Branch, Islamic Azad University, Rasht 41335-3516, Iran; 4 CREA-Research Centre for Food and Nutrition, Via Ardeatina 546, 00178 Rome, Italy; 5 Clinical Unit of Equine Internal Medicine, Veterinarmedizinische Universitat, 1210 Wien, Austria; 6 Department of Pharmacy, University of Napoli Federico II, 80131 Napoli, Italy

The authors would like to make the following corrections to the published paper [1]:

1. A simple summary was added.

2. “*p* < 0.001” was changed to “*p* < 0.01” throughout the paper.

3. In the footnotes of Tables 3–7, the definition of “^a,b^” was changed to “Means within the same row with common superscript letters are not significantly different (*p* ≥ 0.05)”.

4. Some data of the three parameters of broiler chicken growth performance were corrected in Table 3 and, therefore, in the related sentences in the paper. The corrected version is as follows:

The authors would like to apologize for any inconvenience caused to the readers by these changes.

## Figures and Tables

**Table 3:** Growth performance of Ross 308 broilers fed diets containing different levels of L-carnitine and lysine-methionine from day 1 to day 42 of age.

Parameters	Period	Diets									SEM	*p*
		C	D1	D2	D3	D4	D5	D6	D7	D8		
Feed intake, g/pen/period	1–21 d	997.30	1012.60	1009.90	988.67	977.43	983.57	1005.43	1012.30	1006.53	8.37	NS
	22–35 d	2815.30	2898.46	2892.30	2819.50	2810.20	2800.90	2814.60	2859.19	2793.13	50.49	NS
	35–42 d	1851.90 ^a^	1702.40 ^ab^	1781.90 ^ab^	1862.00 ^a^	1748.9 ^ab^	1564.40 ^b^	1945.10 ^a^	1872.80 ^a^	1757.70 ^ab^	83.30	*
	1–42 d	5664.50 ^ab^	5613.46 ^ab^	5684.10 ^ab^	5670.17 ^ab^	5536.53 ^ab^	5348.87 ^b^	5765.13 ^a^	5744.29 ^a^	5557.36 ^ab^	100.06	*
Body weight gain, g/pen/period	1–21 d	697.60 ^ab^	706.31 ^a^	745.50 ^a^	671.10 ^abc^	621.60 ^bc^	610.17 ^c^	733.67 ^a^	741.00 ^a^	723.27 ^a^	18.57	**
	22–35 d	1207.10 ^a^	1241.29 ^a^	1165.50 ^ab^	1066.67 ^c^	1029.83 ^c^	1042.00 ^c^	1115.17 ^bc^	1100.41 ^bc^	1089.90 ^bc^	29.31	**
	35–42 d	935.60 ^b^	898.60 ^b^	917.60 ^b^	889.03 ^b^	949.27 ^b^	950.53 ^b^	1037.36 ^a^	1010.89 ^a^	976.53 ^b^	69.53	*
	1–42 d	2840.30 ^a^	2846.20 ^a^	2828.60 ^a^	2626.80 ^b^	2600.70 ^b^	2602.70 ^b^	2886.20 ^a^	2852.30 ^a^	2789.70 ^a^	86.91	*
Feed Conversion ratio	1–21 d	1.43 ^bc^	1.43 ^bc^	1.35 ^c^	1.47 ^bc^	1.57 ^ab^	1.61 ^a^	1.37 ^c^	1.36 ^c^	1.39 ^c^	0.03	**
	22–35 d	2.33 ^c^	2.33 ^c^	2.48 ^bc^	2.64 ^ab^	2.73 ^a^	2.69 ^a^	2.52 ^ab^	2.60 ^ab^	2.56 ^ab^	0.06	**
	35–42 d	1.98	1.89	1.94	2.09	1.84	1.64	1.88	1.85	1.80	0.09	NS
	1–42 d	1.99 ^ab^	1.97 ^b^	2.01 ^ab^	2.16 ^a^	2.13 ^a^	2.05 ^a^	2.00 ^ab^	2.01 ^ab^	1.99 ^ab^	0.04	*

C (Control) = diet with lysine, methionine, and L-carnitine equal to NRC recommendations; D1 = control diet supplemented with lysine at 15% in excess of NRC, methionine at 15% in excess of NRC, and L-carnitine equal to NRC; D2 = control diet supplemented with lysine at 30% in excess of NRC, at 30% in excess of NRC, and L-carnitine equal to NRC; D3 = control diet supplemented with lysine equal to NRC, methionine equal to NRC, and L-carnitine at 15% in excess of NRC; D4 = control diet supplemented control diet supplemented with lysine at 15% in excess of NRC, methionine at 15% in excess of NRC, and L-carnitine at 15%in excess of NRC; D5 = control diet supplemented lysine at 30% in excess of NRC, methionine at 30% in excess of NRC, and L-carnitine at 15% in excess of NRC; D6 = control diet supplemented with lysine equal to NRC recommendations, methionine equal to NRC recommendations, and L-carnitine at 75% in excess of NRC ; D7 = control diet supplemented with lysine at 15% in excess of NRC, methionine at 15% in excess of NRC, and L-carnitine at 75% in excess of NRC; D8 = control diet supplemented with lysine at 30% in excess of NRC, methionine at 30% in excess of NRC, and L-carnitine at 75% in excess of NRC; SEM = standard error of the mean; * *p* < 0.05, ** *p* < 0.01, NS = *p* ≥ 0.05; a, b: Means within the same row with common superscript letters are not significantly different (*p* ≥ 0.05).

## References

[1] Ghoreyshi S.M., Omri B., Chalghoumi R., Bouyeh M., Seidavi A., Dadashbeiki M., Lucarini M., Durazzo A., van den Hoven R., Santini A. (2019). Effects of Dietary Supplementation of L-Carnitine and Excess Lysine-Methionine on Growth Performance, Carcass Characteristics, and Immunity Markers of Broiler Chicken. Animals.

